# An Artificial Turf-Based Surrogate Surface Collector for the Direct Measurement of Atmospheric Mercury Dry Deposition

**DOI:** 10.3390/ijerph14020173

**Published:** 2017-02-10

**Authors:** Naima L. Hall, Joseph Timothy Dvonch, Frank J. Marsik, James A. Barres, Matthew S. Landis

**Affiliations:** 1 Department of Environmental Health Sciences, School of Public Health, University of Michigan Air Quality Laboratory, Ann Arbor, MI 48109, USA; hallnai@umich.edu (N.L.H.); dvonch@umich.edu (J.T.D.); marsik@umich.edu (F.J.M.); jbarres@umich.edu (J.A.B.); 2 U.S. Environmental Protection Agency Office of Research and Development, Research Triangle Park, NC 27709, USA

**Keywords:** mercury, dry deposition, surrogate surface, turf

## Abstract

This paper describes the development of a new artificial turf surrogate surface (ATSS) sampler for use in the measurement of mercury (Hg) dry deposition. In contrast to many existing surrogate surface designs, the ATSS utilizes a three-dimensional deposition surface that may more closely mimic the physical structure of many natural surfaces than traditional flat surrogate surface designs (water, filter, greased Mylar film). The ATSS has been designed to overcome several complicating factors that can impact the integrity of samples with other direct measurement approaches by providing a passive system which can be deployed for both short and extended periods of time (days to weeks), and is not contaminated by precipitation and/or invalidated by strong winds. Performance characteristics including collocated precision, in-field procedural and laboratory blanks were evaluated. The results of these performance evaluations included a mean collocated precision of 9%, low blanks (0.8 ng), high extraction efficiency (97%–103%), and a quantitative matrix spike recovery (100%).

## 1. Introduction

In recent years, a growing number of intensive field campaigns and routine measurement networks have provided valuable information on the rates of total mercury (Hg) wet deposition in North America [1,2,3,4,5,6,7,8,9,10,11,12,13]. The ability to place bounds on the rates of total Hg dry deposition has been hampered by the relative lack of direct measurement approaches to quantify this critical process for the three most relevant forms of Hg: gaseous elemental Hg (Hg^0^), gaseous oxidized Hg (GOM), and particulate bound Hg (Hg(p)). Initial mercury dry deposition measurement estimates focused on the use of micrometeorological [14,15,16,17,18], dynamic flux chamber [19,20,21], vegetative throughfall [2,22,23,24,25,26], and inferential modeling approaches [4,27,28,29,30,31,32]. The application of micrometeorological approaches, such as modified Bowen ratio and relaxed eddy accumulation, are typically focused on just the gaseous Hg species, can be challenging to use in remote areas as they require a stable source of electrical power, and stringent site selection criteria must be adhered to insure adequate uniform upwind fetch [33]. Dynamic flux chambers also require a stable source of electrical power, and can disrupt the natural temperature, humidity, and turbulent field responsible for the fluxes they are attempting quantify [18,34]. Vegetative throughfall approaches require an amenable canopy, sampling intervals are limited by the frequency of rain events, and spatial investigation is complicated by unique canopy structure and dry deposition collection characteristics of the canopy above each sampling location. Inferential modeling approaches rely on parameterizations of land surfaces, estimates of meteorological conditions and turbulence, ambient Hg measurements, and estimates of representative Hg(p) mass median aerodynamic diameter (MMAD).

Surrogate surface (e.g., water, filter, greased Mylar film) approaches have also been utilized for direct measurement of Hg dry deposition [24,30,35,36,37,38,39,40,41,42,43,44]. While each of the aforementioned approaches were successfully utilized for the measurement of Hg dry deposition, each method has limitations with respect to widespread, long-term deployment, and application. Surrogate surface approaches often suffer from the need to be visually monitored and attended, such that the surfaces avoid potential contamination from precipitation (wet deposition). Additional complications include the impact of strong winds, which can cause a loss of sample solution in the case of water surrogate surfaces, and evaporation which disrupts the even surface across the surface and changes laminar flow. While some engineering approaches have been incorporated into automated water surrogate surface collectors to minimize evaporative water loss [45] and loss of sample solution due to wind [46], the cost, power requirements, and region specific meteorological conditions may restrict the broad application of these solutions.

This manuscript describes the development and evaluation of an artificial turf surrogate surface (ATSS) methodology for the measurement of total Hg dry deposition. The ATSS has been developed to overcome many of the aforementioned limitations of other dry deposition measurement techniques. The ATSS is a passive surrogate surface approach that utilizes a three-dimensional deposition surface that more closely mimics the physical structure of many natural surfaces than traditional flat surrogate surface designs. The design of the ATSS allows for the surface to be deployed in the field for either short or extended periods of time (days to a weeks) without risk of contamination from rainfall because the system has been designed to measure total (wet and dry) Hg deposition. Mercury dry deposition to the ATSS is calculated by determining the difference between the total Hg deposition measured by the ATSS and a collocated measure of wet-only total Hg deposition.

## 2. Artificial Turf Surrogate Sampler Design

**Figure 1:**
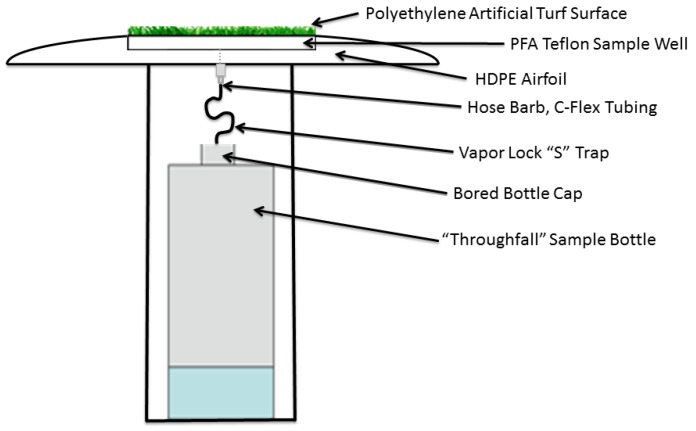
Schematic of Artificial Turf Surrogate Surface (ATSS) sampler.

The ATSS (Figure 1; Figure S1a) was modified from a static water surrogate surface (SWSS) configuration (Figure S1b) previously used by Marsik et al. [30] and initially described by Keeler et al. [7]. The ATSS consists of a high density polyethylene (HDPE) Frisbee-shaped aerodynamic laminar flow airfoil designed to collect dry-depositing gases and particles without altering the existing turbulent air flow field [47]. The ATSS airfoil contains a removable 19 cm outer diameter Teflon Perfluoroalkoxy alkane (PFA) sample plate insert that holds an 18 cm diameter circular piece of artificial turf that is mechanically punched from polyethylene AstroTurf^®^ NXT (GrassWorx, St. Louis, MO, USA). The gridded turf backing consists of a matrix of open spaces allowing precipitation to flow through the turf and into a “throughfall” collection bottle.

The aerodynamic airfoil is attached to a 30 cm length of 10 cm diameter Polyvinyl Chloride (PVC) base unit using sheet metal screws via an attachment surface located under the bored well. The custom PVC base is bolted to a fabricated aluminum support bracket, and mounted vertically to an anchored tripod. Once the Teflon PFA plate is inserted into the airfoil well, a Teflon PFA hose barb attachment is threaded into the bottom. A 5 cm length of C-Flex thermoplastic elastomer tubing on either side of a glass “p-trap” vapor lock is attached to the Teflon PFA collection plate hose barb fitting. The other end of the tubing is fed through a bored cap into a 1 L fluorinated high density polyethylene (HDPE) throughfall collection bottle (Nalgene, Rochester, NY, USA, #2097-0032). The enclosed PVC support base has a small acrylic beam to support the weight of the bottle during sample collection, and a removable aluminum cover is installed to shield the sample from sunlight. The ATSS airfoil is deployed at a height approximately 1–2 m above the ground in an area of clear fetch, depending upon the nature of the terrain and the average height of the surrounding vegetation, to insure uniformity of air flow and to insure that precipitation splash from any near-field objects did not enter the sampler.

Following deployment, the ATSS was left exposed to ambient air for a predetermined sampling period (typically three or four days for this study) after which the exposed turf, throughfall, and collocated wet deposition samples were collected and returned to the laboratory for analysis. The total Hg dry deposition collected by the ATSS was determined using Equation (1).

(1) $$Total\;Dry\;Deposition\;\left(ng\cdot m^{-2}\cdot h^{-1}\right)=\frac{\left(Turf+Throughfall\right)-Wet\;Deposition\;Contribution}{Collection\;Surface\;Area\times Turf\;Exposure\;Time}$$

where

*Turf* = turf extract concentration × volume of extraction solution;
*Throughfall* = throughfall concentration × sample volume;
*Wet Deposition Contribution* = volume weighted average precipitation concentration × (throughfall sample volume—rinse solution volume); and
*Collection Surface Area* = 0.025687232 m^−2^.

## 3. Methods

### 3.1. Site Description

The ATSS method development and performance evaluations were conducted during three field measurement intensive studies in Michigan and Florida.

#### 3.1.1. Michigan Studies

The initial ATSS field evaluation study was conducted at two urban sites in southeast Michigan, in Dearborn (42.3075° N, 83.1496° W) and in Detroit-Fort Street (42.3026° N, 83.1067° W). The Dearborn and Detroit sites were located at existing Michigan Department of Environmental Quality air monitoring sites. Sample collection was conducted during 18 July–8 August 2007. A few of the ATSS samplers at Dearborn were deployed in duplicate to determine collocated precision.

Additional field evaluations were performed during 4 August–13 September 2008, as part of a multi-institutional Hg dry deposition measurement inter-comparison study. This study was performed at the University of Michigan Matthaei Botanical Gardens in Ann Arbor, MI (42.2987° N, 83.6647° W). The ATSS samplers at the Botanical Gardens were deployed in duplicate to determine collocated precision.

#### 3.1.2. Florida Study

After cleaning method refinements, further evaluations of sampler blank performance were conducted at monitoring sites in Tampa and Davie, Florida (27.9134° N, 82.3752° W; 26.0854° N, 80.2407° W, respectively) from 4 July to 4 August 2010, and in Jacksonville, Florida (30.2475° N, 81.9510° W) from 25 July to 24 August 2010. While the ATSS Michigan studies were developed to measure Hg and trace element (not discussed here) dry deposition using a single surface, the Florida studies adopted expanded cleaning and sampling protocols to measure Hg dry deposition in one collector, and major ion and trace element dry deposition in a separate collocated ATSS sampler. The cleaning and sampling protocols below describe the finalized method used in the Florida studies.

### 3.2. Cleaning Procedure

Prior to use in the field, a multi-step acid cleaning procedure was performed on all supplies that were in contact with samples. The exact procedure followed varied by component of the sampling system. The turf surfaces were soaked in 3.5% *v*/*v* HNO_3_ for five days, subsequently rinsed five times in ASTM type I (18.2 MΩ∙cm) water, and then air-dried in a HEPA-filtered cabinet. The cleaning procedure applied for the turf surfaces punches used in Florida (Tampa, Davie and Jacksonville, FL, USA) added an additional 24-h soak in 1% BrCl solution after the HNO_3_ soak, then were rinsed five times in ASTM type I water and dried in a HEPA-filtered air cabinet. The Teflon wells, Teflon hose barb adapters and glass vapor locks were soaked for six hours in a solution of 3.5% *v*/*v* HNO_3_ at 70 °C, then were rinsed five times in ASTM type I water and dried in a HEPA-filtered cabinet. C-flex tubing was soaked in 3.5% *v*/*v* HNO_3_ for 24 h before it was rinsed five times with ASTM type I water and dried in a HEPA-filtered air cabinet. All fluorinated HDPE sample and turf extraction bottles were acid-cleaned using the method described in Landis and Keeler [48]. Following drying, all sampling supplies were triple-bagged in sealable polyethylene bags to insure cleanliness during transportation to and from the field measurement sites.

### 3.3. Sample Deployment 

Ultra-clean field handling techniques were employed to avoid contamination of samples. Site operators wore particle-free vinyl clean-room gloves for sample deployment, used acid-cleaned Teflon-coated forceps when handling turf, and always stood downwind from the sampling media. Before each ATSS and SWSS sample deployment, airfoils were wiped with particle-free polypropylene clean-room wipes and ASTM type I water, and all other acid-cleaned sampling components were replaced after each sample collection. At the end of each sampling period, the site operator placed the turf surface into a 2 L wide-mouth fluorinated HDPE bottle, then rinsed the turf sample well with 30 mL 2.2% *v*/*v* HNO_3_ solution to insure that residual dry deposition was washed off into the throughfall collection bottle. Field blanks were conducted by deploying clean sampling supplies, including a clean turf surface and throughfall bottle, and then pouring approximately 300 mL of ASTM type I water through the turf. The field blank turf and throughfall were then collected following the same protocols as described for sample collection. Collocated wet-only precipitation samples were collected using a modified MIC-B method described by Landis and Keeler [4,48].

### 3.4. Sample Extraction and Analysis

Samples were stored in a dark cold room (4 °C) before and after processing prior to analysis. The ATSS throughfall and collocated modified MIC-B wet-only precipitation samples were oxidized to 1% (*v*/*v*) solution with concentrated BrCl in a class 100 clean room at least 24-h prior to analysis [8]. Turf surfaces were extracted in the 2 L bottles by adding 350 mL of ASTM type I water and immediately oxidizing with concentrated BrCl to a 4% solution (*v*/*v*). Bottles were then turned while in an ultra-sonic water bath for 2 h. All Hg sample analysis was conducted using a Tekran Instruments Corporation, (Knoxville, TN, USA) Model 2600 Cold Vapor Atomic Fluorescence Spectroscopy (CVAFS) analyzer following EPA Method 1631e.

### 3.5. Data Analysis

Data processing and descriptive statistics were performed using SAS v.9.4 (SAS Institute, Cary, NC, USA). The normality of the ATSS data distributions were examined using skewness and kurtosis coefficients, and the Shapiro–Wilk test. Normality tests and the Brown–Forsythe test for equal variance were used to evaluate the assumptions of parametric procedures.

## 4. Results and Discussion

### 4.1. Performance Characteristics

#### 4.1.1. Field Blanks

The addition of the 24-h BrCl soak to the turf cleaning method during the Florida studies (described in Section 3.2) resulted in a significant decrease in the mass of Hg in blank samples. Two different cleaning methods were used during the testing and deployment of this method. Consequently, the blank data from the Michigan monitoring sites was analyzed separately from blanks collected during the Florida studies.

Six ATSS surface field blank samples were collected during the initial Michigan studies (Table 1) with a mean total Hg mass of 2.0 ± 1.1 ng, which was equivalent to a total mean Hg deposition of 76 ± 43 ng·m^−2^, and represented 18% of the measured Hg mass over a typical sample period. The total field blank mass was a sum of the Hg extracted from the ATSS surface plus the Hg from the throughfall blank and the acid solution. The average contribution of the throughfall blank toward the total blank was insignificant at 0.03 ng (<0.1%). After quantifying the relatively large field blank contribution of the ATSS surface during the early Michigan studies, the ATSS surface cleaning method was modified to include a 24-h soak in 1% BrCl, and the Michigan studies results were field-blank corrected using their site specific values.

After the new cleaning methodology was implemented, the average ATSS field blank mass of total Hg collected during the Florida studies (*n* = 77) was significantly reduced (*p* < 0.05) to 0.77 ± 0.44 ng (Table 2). Approximately 80% (61 out of the 77) of the ATSS field blanks were below the analytical detection limit. While improvements in the cleaning method led to a drastic reduction in the ATSS field blank mass, the blank turf mass still contributed on average 23% of the total Hg dry deposition measured over a typical three-day ATSS sample period at the Florida sites due to correspondingly lower deposition rates in Florida. As a result, the Florida ATSS results were also field blank corrected.

#### 4.1.2. Extraction Efficiency

ATSS extraction tests were conducted to evaluate the efficiency of the extraction method. NIST SRM 1633b, (Constituent Elements in Coal Fly Ash) and 2704 (Buffalo River Sediment) were used to approximate Hg(p). The powders were weighed out and distributed onto an ATSS turf sampling surface. Extraction and analytical methods described above were followed. Analytical results were field-blank corrected with a control turf processed and analyzed concurrently. The field blank value was 1.78 ng which was comparable with the Hg mass from the Michigan study field blanks. The mean percent recovery using the SRM 1633b was 97% ± 10% (*n* = 3) and the mean percent recovery SRM 2704 samples was 103% ± 1% (*n* = 2).

Matrix spikes were also conducted on the extracted turf samples throughout all study analyses using 250 μL addition of 2 mg·mL^−1^ Hg solution. The percent recovery of matrix spikes (*n* = 28) was 100% ± 4% (mean ± standard deviation), with a minimum of 91% and a maximum of 110%.

#### 4.1.3. ATSS Total Dry Deposition Partitioning

In the absence of precipitation during an ATSS collector sampling period, the total dry deposition is effectively calculated as the sum of the turf and the acid rinse of the turf well into the throughfall collection bottle Equation (1). During this study, we found in the absence of rain (*n* = 14) the percentage of the total Hg dry deposition collected onto the turf media was 98.3% ± 1.5% (mean ± standard deviation) suggesting a small amount of Hg(p) collected by the turf migrated down into the turf well (Table S1). For ATSS samples exposed to rain events (*n* = 15) the percentage of the total Hg dry deposition remaining on the turf media was 65.1% ± 11.9% demonstrating the translocation of dry deposition into the throughfall collection bottle. The variability in the amount of Hg mass collected on the ATSS turf media in rain exposed samples was a function of when during the sampling period the precipitation event occurred and the intensity of the event. The Hg mass collected into the throughfall collection bottle originating from the rain itself was subtracted (Equation (1)) based on the volume of ATSS throughfall collected and the volume weighted average concentration [4] of the associated collocated wet-only event precipitation sample(s).

#### 4.1.4. ATSS Collocated Precision

**Figure 2:**
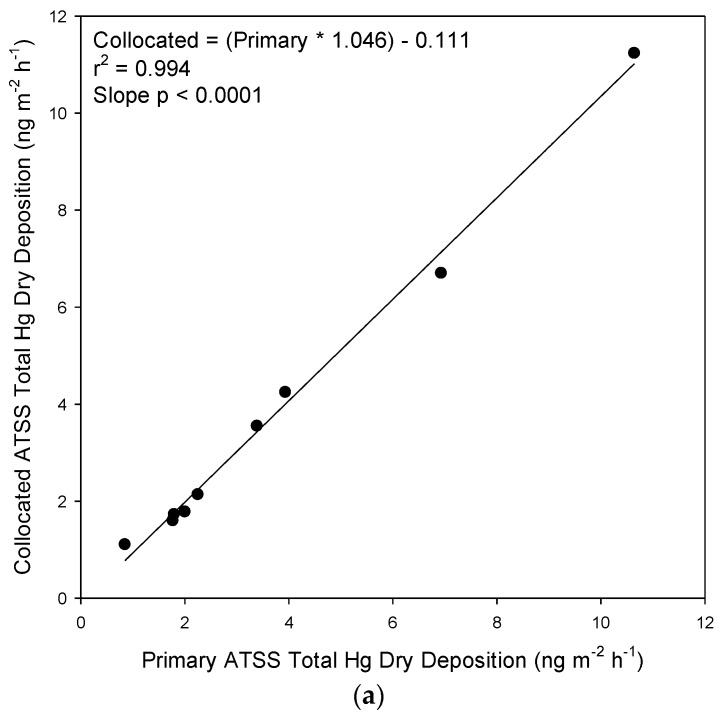

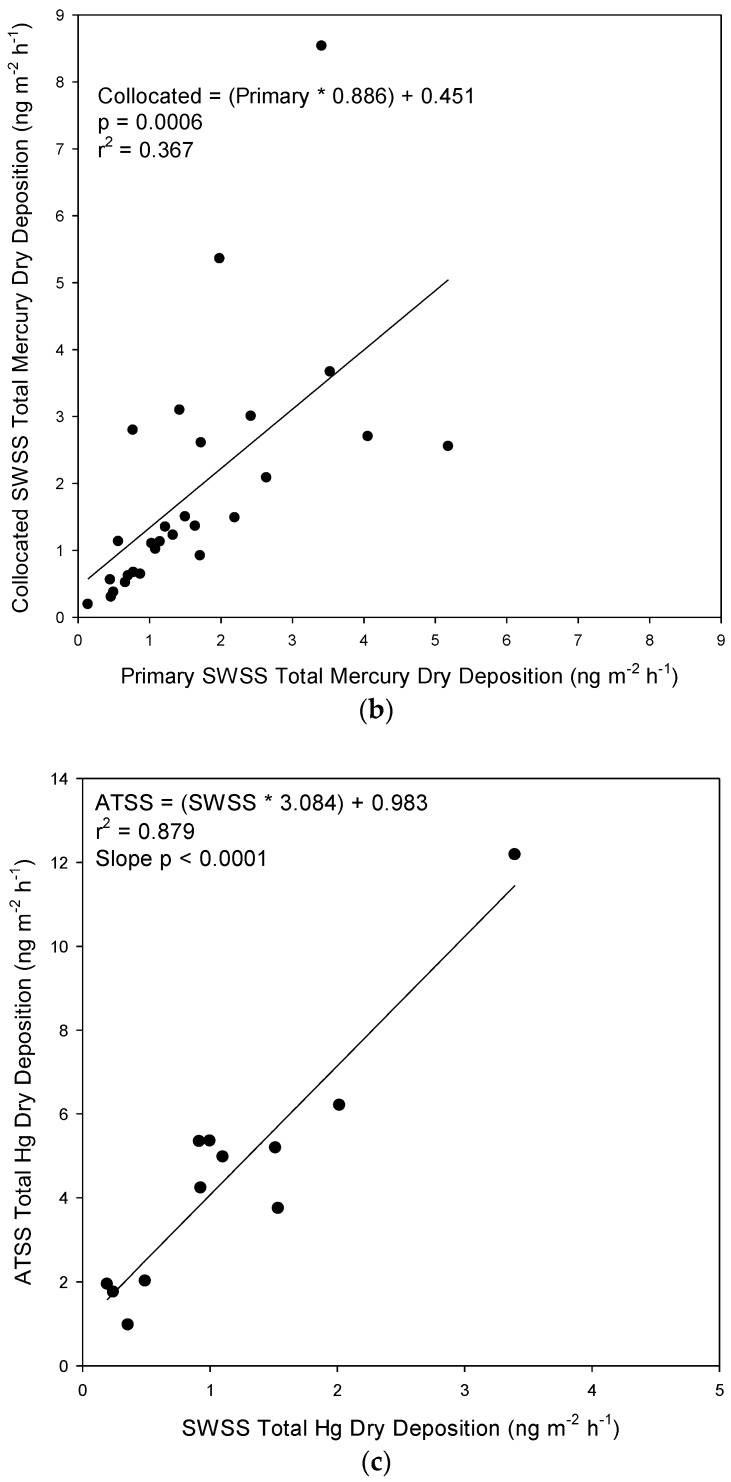
(**a**) Collocated Artificial Turf Surrogate Surface (ATSS) results; (**b**) Collocated Static Water Surrogate Surface (SWSS) results; and (**c**) Artificial Turf Surrogate Surface (ATSS) versus the mean of collocated Static Water Surrogate Surface (SWSS) Collector results for total Hg Dry deposition results.

The precision of the ATSS method was evaluated by deploying collocated samplers. The results from the collocated samplers were not significantly different for Hg dry deposition flux, evaluated by a dependent paired t-test modeling the primary collector against the collocated collector for any of the sites in Michigan (Figure 2a). The absolute percent difference (APD) for the individual sampling periods (*n* = 9) ranged from 3.3% to 26.7%. The mean APD between collocated ATSS samplers was 8.7%, and the median APD was 5.4%. Based on the Figure 2a regression slope, the average difference between the primary and collocated samplers was ~5%.

#### 4.1.5. SWSS versus ATSS Comparison

Collocated SWSS collectors were deployed alongside the ATSS collector at the Dearborn site to compare the performance of the new ATSS collector design to the more routinely used water collector configuration. Our first observation was that the collocated precision of the SWSS collectors (*n* = 28) was significantly worse than the ATSS collectors with a mean APD of 35% and a median APD of 25% (Figure 2b). The mean of the collocated SWSS collectors total Hg dry deposition results were used to compare with the ATSS measurements. The ATSS collector was found to measure systematically higher total Hg dry deposition flux than that from the SWSS sampler due to the greater bladed turf collection surface (Table 3), but the two methods were highly correlated (Figure 2c) with a coefficient of determination of 0.879. On average, the ATSS collector provided results that were a factor of ~3 higher than the SWSS collector. Accounting for the average height, width, and density of the blades from turf surfaces we estimate an effective three-dimensional surface area of 0.131 m^2^, approximately five times greater than the simple two-dimensional surface area calculations for the water surfaces (0.0257 m^2^). The observation that the actual measured total Hg dry deposition flux enhancement (~3 times) was lower than the increased collection surface area (~5 times) is consistent with the published literature on real world vegetative edge effects and lower atmospheric deposition of in stand atmospheric deposition [49,50,51,52], and the observed importance of the physical structure and geometry of wild grasses as some species have higher deposition velocities despite lower leaf area indexes [53].

The comparison between the ATSS and SWSS collector total Hg fluxes also need to be considered in light of SWSS samples that were invalidated due to contamination by wet deposition events. The ATSS method reflects deposition during a continuous exposure period (less the period of precipitation events), while the SWSS samplers were only deployed for 12- or 24-h durations. Any precipitation during the SWSS sampling period invalidated the entire sample representing up to 24 h of deposition, during which time the ATSS sampler was deployed and collecting. Consequently, during sampling periods with precipitation the valid collection time of the SWSS collectors were reduced to 50%–75% of the collocated ATSS collector. This comparison highlights several benefits of the ATSS sampler design: (i) the ATSS collects a higher mass loading increasing Hg detectability; (ii) the ATSS method is more precise; (iii) the ATSS samplers can be deployed for longer unattended periods; and (iv) the ATSS method provides higher temporal coverage since periods of wet deposition do not invalidate the samples.

The ATSS and SWSS fluxes measured during these studies were similar to what has been measured in this region during other studies. Liu et al. [54] measured an average dry deposition with the same SWSS method at a Detroit site of 3.3 ng·m^−2^·h^−1^ with a median of 1.2 ng·m^−2^·h^−1^ and Gildmeister [55] measured Hg dry deposition fluxes at Detroit sites between 0.4 and 1.4·ng·m^−2^·h^−1^, using greased Mylar strips. Another difference between the suburban Botanical Gardens site and the urban/industrial Dearborn and Detroit-Fort Street sites was the mean concentration of GOM measured using the method described by Landis et al. [56] was approximately five times higher at the Dearborn (23 pg·m^−3^) and Fort Street sites (27 pg·m^−3^). These GOM levels are similar to those previously reported at nearby Detroit sites by Liu et al. [54,57].

### 4.2. Urban Gradients and the Relative Importance of Hg Dry Deposition

**Figure 3:**
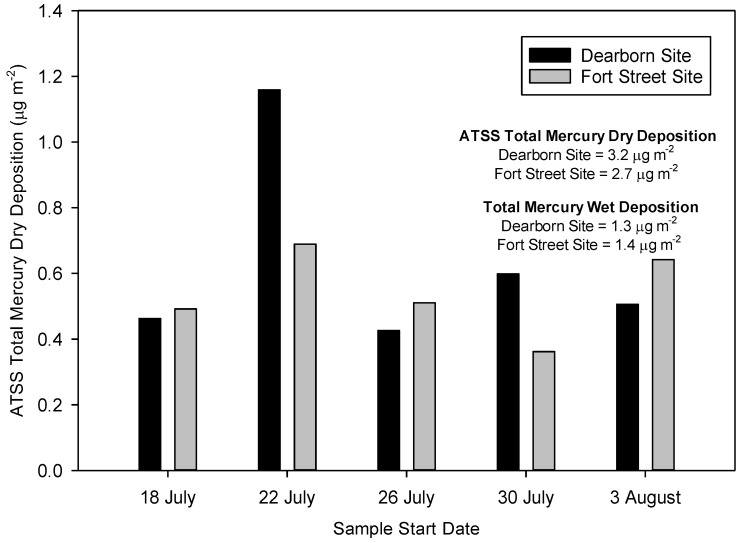
Spatial Evaluation of Artificial Turf Surrogate Surface (ATSS) Total Mercury Dry Deposition Results Between Detroit and Dearborn, MI Monitoring Sites.

The ATSS data from the Dearborn study also provides insight into the potential impact of coarse particle Hg(p) dry deposition and the relative importance of wet and dry deposition in an urban/industrial area. The concurrent ATSS measurements at the Dearborn and Detroit-Fort Street sites are presented in Figure 3. The distance between Dearborn and Detroit-Fort Street sampling sites is only 3.6 km, yet the total Hg dry deposition results for the ATSS samples starting on 22 July (1.16 μg·m^−2^ versus 0.69 μg·m^−2^) and 30 July (0.60 μg·m^−2^ versus 0.36 μg·m^−2^) were substantially different. The magnitude of the observed differences in total Hg dry deposition over such short spatial scales suggests the influence of coarse fraction aerosols, which typically show more spatial heterogeneity in urban areas [58,59] and have higher associated deposition velocities [4,60]. Total Hg wet deposition observed at the Dearborn (1.3 μg·m^−2^) and Detroit (1.4 μg·m^−2^) sites were virtually identical over the study period. The dry deposition of total Hg was found to be higher than total Hg wet deposition at the urban/industrial Dearborn and Detroit-Fort Street sites by a factor of 2.5 and 1.9, respectively.

## 5. Conclusions

The ATSS collector was found to be an effective new tool for the direct quantification of atmospheric total Hg dry deposition. The ATSS collector overcomes the major shortcomings identified with previous direct surrogate measurement approaches such as invalidation of samples impacted by precipitation or strong winds. The three-dimensional surface of the ATSS collector more realistically simulates natural vegetative surfaces, and results in the collection of additional Hg mass that decreases the analytical detection limit. Spike recoveries, collocated evaluations, and comparisons to SWSS collectors demonstrate that the ATSS collector is both accurate and precise.

The ATSS collector is ideally suited for investigating Hg dry deposition over both short collection times (day(s)) typically preferred during short-term intensive research studies, as well as longer collection times (week(s)) typical of long-term routine network monitoring when site operators may only be scheduled to visit a sampling site once a week. Longer deployment times would minimize the blank contribution, reduce method detection limits, and may increase the precision of the samples. Whereas frequent high volume rainfall events are common in Florida during the summer, in Michigan, the throughfall bottles could be deployed for up to seven days. Samplers could also be modified to collect throughfall into 2 L bottles, thus eliminating a more frequent need to collect and change the sample bottles.

## Figures and Tables

**Table 1:** Summary of total Hg ATSS field blanks at Michigan sites (ng).

Blank Metric	Throughfall	Turf	Total
*n*	6	6	6
Mean	0.03	1.96	1.99
Standard Deviation	0.02	1.12	1.12
Minimum	0.01	0.52	0.56
Maximum	0.05	3.07	3.09
Median	0.03	2.31	2.33

**Table 2:** Summary of total Hg ATSS field blanks at Florida sites (ng).

Blank Metric	Throughfall	Turf	Total
*n*	77	77	77
Mean	0.07	0.77	0.84
Standard Deviation	0.07	0.44	0.44
Minimum	0.01	0.12	0.19
Maximum	0.39	1.96	2.01
Median	0.05	0.71	0.75

**Table 3:** Comparison of Artificial Turf Surrogate Surface (ATSS) versus Static Water Surrogate Surface (SWSS) sampler at Michigan Sites.

Site	*n*	ATSS Flux (ng·m^−2^·h^−1^)	SWSS Flux (ng·m^−2^·h^−1^)
Botanical Gardens	3	1.56	0.26
Dearborn	5	6.42	2.22
Detroit-Fort Street	5	5.38	1.29

## Supplementary Materials

The following are available online at www.mdpi.com/1660-4601/14/2/173/s1, Figure S1: Pictures of a Deployed Artificial Turf Surrogate Surface (ATSS) collector surface (a); and a Static Water Surrogate Surface (SWSS) collector surface (b). Table S1: Summary of Artificial Turf Surrogate Surface (ATSS) samples collected at the University of Michigan Botanical Gardens (BOT), Dearborn (DBN), and Detroit-Fort Street (FRT) air monitoring sites.
