# Supplementary material for: An Artificial Turf-Based Surrogate Surface Collector for the Direct Measurement of Atmospheric Mercury Dry Deposition
Source: Int J Environ Res Public Health. 2017 Feb 10;14(2):173. doi: 10.3390/ijerph14020173 (PMC5334727; doi:10.3390/ijerph14020173)
Supplement: Supplementary file 1 [file ijerph-14-00173-s001.pdf]

Supplementary Materials: An Artificial Turf-Based Surrogate Surface Collector for the Direct Measurement of Atmospheric Mercury Dry Deposition

Naima L. Hall, Joseph Timothy Dvonch, Frank J. Marsik, James A. Barres and Matthew S. Landis

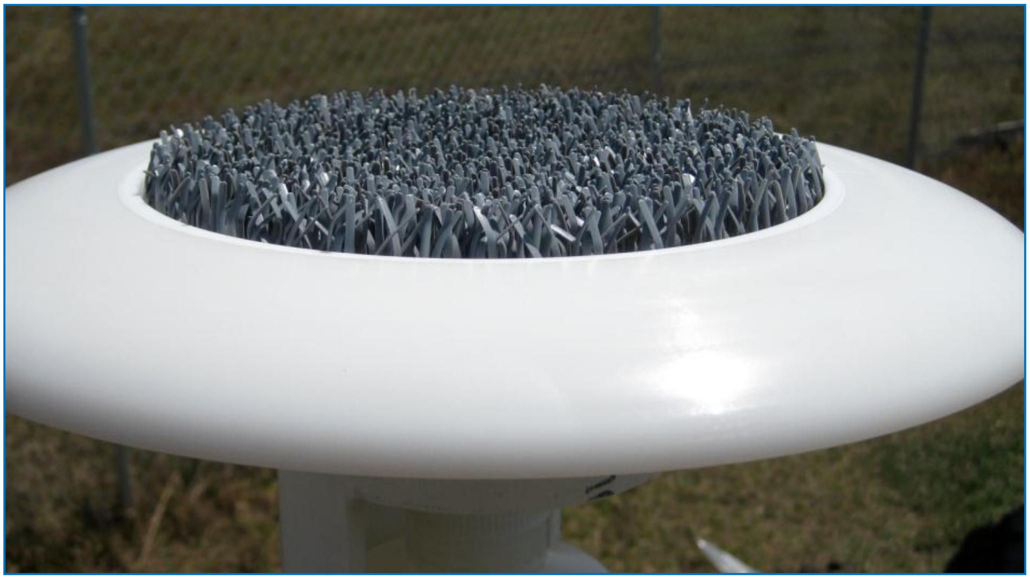

(**a**)

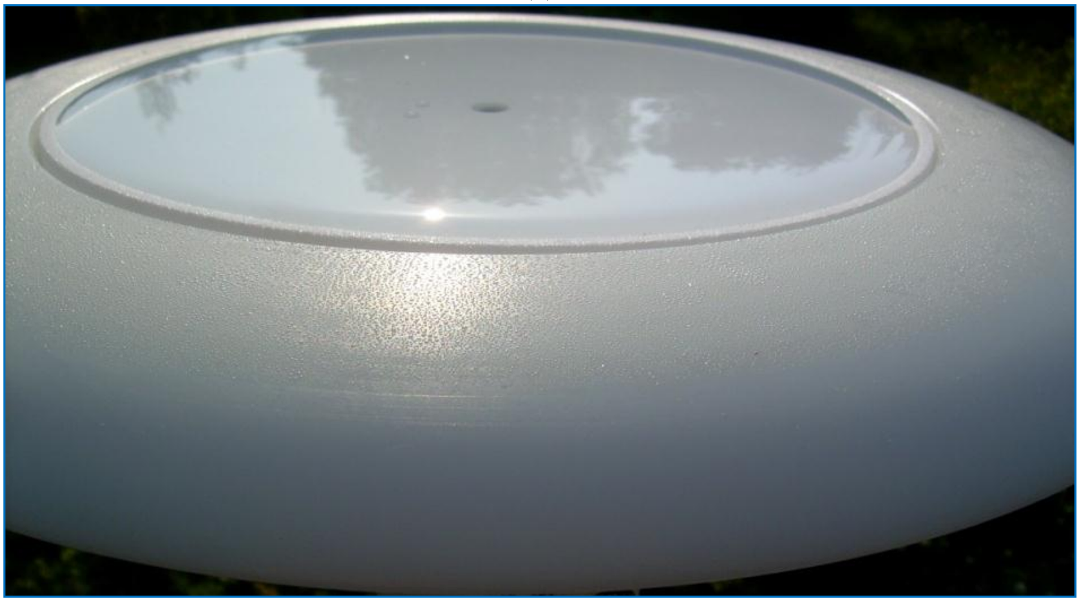

(**b**)

Figure S1. Pictures of a Deployed Artificial Turf Surrogate Surface (ATSS) collector surface (**a**); and a Static Water Surrogate Surface (SWSS) collector surface (**b**).

Table S1. Summary of Artificial Turf Surrogate Surface (ATSS) samples collected at the University of Michigan Botanical Gardens (BOT), Dearborn (DBN), and Detroit-Fort Street (FRT) air monitoring sites.

Site	Sample	Start Date	Duration (min)	Total Dry Deposition ($ng{\cdot}m^{-2}{\cdot}h^{-1}$)	Turf Dry Deposition ($ng{\cdot}m^{-2}{\cdot}h^{-1}$)	Throughfall Contribution ($ng{\cdot}m^{-2}{\cdot}h^{-1}$)	Wet Deposition Subtracted ($ng{\cdot}m^{-2}{\cdot}h^{-1}$)	Amount of Total Dry Deposition Collected on Turf (%)
**BOT**	1	4 August 2008	4345	1.79	1.77	0.02	0	98.6
	1C *	4 August 2008	4330	1.73	1.71	0.02	0	98.7
	2	7 August 2008	703	10.64	6.41	4.36	0.13	60.2
	2C *	7 August 2008	696	11.24	6.76	4.62	0.15	60.2
	3 §	7 August 2008	3629	1.95	1.89	0.06	0	97.0
	4	10 August 2008	4272	0.85	0.81	0.03	0	96.2
	4C *	10 August 2008	4270	1.11	1.08	0.03	0	97.4
	5	13 August 2008	10,089	1.77	1.75	0.01	0	99.2
	5C *	13 August 2008	10,084	1.60	1.59	0.01	0	99.5
	6	20 August 2008	10,079	2.00	1.99	0.01	0	99.5
	6C *	20 August 2008	10,083	1.78	1.77	0.01	0	99.3
	7	27 August 2008	10,134	2.25	2.24	0.01	0	99.6
	7C *	27 August 2008	10,132	2.14	2.13	0.01	0	99.5
**DBN**	1	18 July 2007	5597	4.95	3.33	1.68	0.06	67.3
	2	22 July 2007	5712	12.16	10.44	1.79	0.07	85.8
	3	26 July 2007	2874	6.93	4.78	2.28	0.13	69.0
	3C *	26 July 2007	2887	6.70	5.04	1.76	0.10	75.2
	4	28 July 2007	2855	1.99	1.88	0.11	0	94.5
	5	30 July 2007	5797	6.19	6.13	0.06	0	99.0
	6	3 August 2007	7187	4.22	2.54	1.77	0.09	60.2
**FRT**	1	18 July 2007	5704	5.17	3.99	1.22	0.05	77.2
	2	22 July 2007	5728	7.21	4.07	3.26	0.12	56.5
	3	26 July 2007	5748	5.32	4.61	0.75	0.05	86.8
	4	30 July 2007	5818	3.73	3.69	0.04	0	98.8
	5	30 August 2007	7209	5.34	3.21	2.22	0.09	60.0
	6	10 August 2007	14,497	3.39	2.14	1.27	0.03	63.3
	6C *	10 August 2007	14,509	3.55	2.08	1.50	0.03	58.7
	7	20 August 2007	10,095	3.93	1.89	2.13	0.08	48.0
	7C *	20 August 2007	10,103	4.25	2.03	2.30	0.08	47.7

Total dry deposition was calculated using Equation (1), and shaded rows denote samples impacted by precipitation events when wet deposition contributions were subtracted. * Denotes collocated samples; § The collocated sample was invalidated due to field site operator error.
